# The Many Facets of Metzincins and Their Endogenous Inhibitors: Perspectives on Ovarian Cancer Progression

**DOI:** 10.3390/ijms19020450

**Published:** 2018-02-02

**Authors:** Ruth M. Escalona, Emily Chan, George Kannourakis, Jock K. Findlay, Nuzhat Ahmed

**Affiliations:** 1 Department of Obstetrics and Gynaecology, University of Melbourne, Parkville, VIC 3052, Australia; ruthescalona74@gmail.com (R.M.E.); epchan@student.unimelb.edu.au (E.C.); jock.findlay@hudson.org.au (J.K.F.); 2 The Hudson Institute of Medical Research, Clayton, VIC 3168, Australia; 3 Fiona Elsey Cancer Research Institute, Ballarat, VIC 3353, Australia; George@fecri.org.au; 4 Federation University Australia, Ballarat, VIC 3010, Australia

**Keywords:** metzincins, ovarian cancer, metastasis, matrix metalloproteinases (MMPs), disintegrin and metalloproteinases (ADAMs), ADAM proteases with thrombospondin motifs (ADAMTS), TIMPs

## Abstract

Approximately sixty per cent of ovarian cancer patients die within the first five years of diagnosis due to recurrence associated with chemoresistance. The metzincin family of metalloproteinases is enzymes involved in matrix remodeling in response to normal physiological changes and diseased states. Recently, there has been a mounting awareness of these proteinases and their endogenous inhibitors, the tissue inhibitors of metalloproteinases (TIMPs), as superb modulators of cellular communication and signaling regulating key biological processes in cancer progression. This review investigates the role of metzincins and their inhibitors in ovarian cancer. We propose that understanding the metzincins and TIMP biology in ovarian cancer may provide valuable insights in combating ovarian cancer progression and chemoresistance-mediated recurrence in patients.

## 1. What Are Metzincins?

Metzincins are one of the five members of zinc endopeptidases (serine, metallo, threonine, aspartic, cysteine) that belong to the metalloproteinase family and are named metzincins due to their conserved Met residue at the active site and the use of a zinc ion in the enzymatic reaction. This family is comprised of Matrixin (MMPs), Adamalysin (ADAMs and ADAMTS), Astacin (Meprin and BMP1/Tolloids), Pappalysins and Serralysin [1,2]. Matrixin and Adamalysin are mostly soluble proteins that are secreted or sequestered by direct binding through the transmembrane domain of many cell types. They play a role in normal tissue remodeling, and are up-regulated in diverse human diseases and cancer [2]. In this review, we will focus on the roles of Matrixin, Adamalysin and their endogenous inhibitors in ovarian cancer.

## 2. The Origin of Epithelial Ovarian Cancer

Ovarian cancer is the eighth most common cancer among women, but is the fifth leading cause of cancer-related death, and is the deadliest of gynecologic cancers [3]. Ovarian cancer was diagnosed in nearly 225,000 women world-wide, and was responsible for estimated 140,100 deaths in 2017 [4]. Nine out of ten women with ovarian cancer have an epithelial ovarian cancer and approximately 70% of these are serous carcinomas. Until recently, the vast majority of epithelial ovarian cancer was thought to arise from the malignant transformation of the ovarian surface epithelium [5,6]. Recently however, high-grade serous ovarian carcinomas are believed to arise from the fimbria of the Fallopian tube [7,8,9], with approximately 90% thought to be derived from the secretory epithelial cells of the Fallopian tube [8]. The development of serous tubal-intra-epithelial carcinoma (STIC), resulting from the acquisition of a *P53* mutation in the distal oviduct fimbriae cells has been proposed to be the origin of ovarian tumors [9,10,11]. This theory is supported by the fact that most ovarian cancers are associated with the presence of STIC, and to-date no premalignant lesions of ovarian cancer have been identified [11]. In addition, the high frequency of *P53* mutations in STICs and high-grade serous carcinoma further supports this perception [11].

A number of studies have suggested malignant transformation of adult ovarian stem cells as a potential cause for the initiation of ovarian cancer [12,13,14,15]. Recently, an ovarian cancer stem cell niche was identified which constituted of the ‘hilum region’, a junctional area between ovarian surface epithelium and oviductal epithelium [14]. The hilum region was shown to contain a population of slow proliferating cells that expressed markers for progenitor cells and demonstrated a predisposition to undergo malignant transformation. In addition, somatic stem cell and stem/progenitor cell characteristics have been reported in ovarian surface epithelium [16,17]. The genes and pathways shown to be associated with adult stem cells were also been shown to be expressed in ovarian surface epithelium [18], suggesting the ovarian surface epithelium may be the potential originator of ovarian cancer. The isolation of multipotent mesenchymal stem cells from the Fallopian tubes has recently been described [19], and the phenotypic characteristics of ovarian cancer cells have been reported in ex vivo cultures of primary Fallopian tube epithelium [20]. These studies suggest both ovarian surface epithelium and tubular epithelium of the Fallopian tube may be the potential origins of ovarian cancer stem cells. An elegant study has recently summarized the current knowledge about ovarian surface epithelium and Fallopian tubal stem cells [21].

## 3. Recent Classification of Epithelial Ovarian Cancer

**Figure 1:**
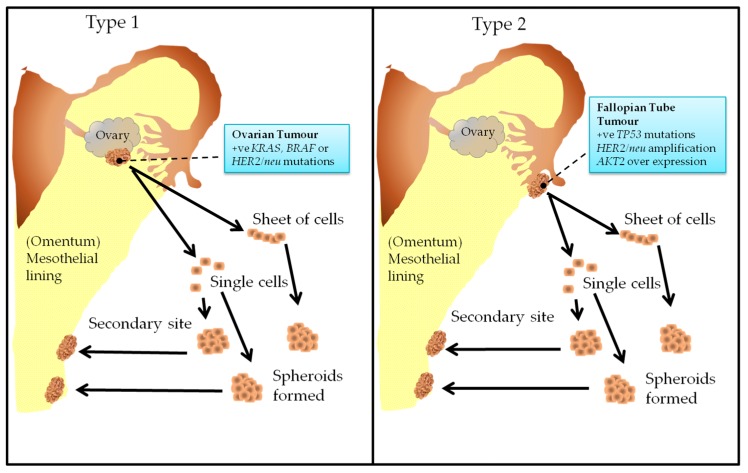
Classification of Epithelial Ovarian Cancer: Type 1: Tumors are thought to evolve from the surface epithelium and the Müllerian inclusions of the ovary. Type 2: Tumors are thought to evolve from the intra-epithelium of the Fallopian tube.

More recently, the classification of epithelial ovarian cancers has been simplified and defined on the basis of two distinctive molecular pathways, and thus have distinct biological behaviors and different cells of origin [22,23]. Type 1 carcinomas are slow growing and are thought to derive from well-defined tumor lesions on ovarian surface epithelium and Müllerian inclusions [23]. They are characterized molecularly by *KRAS*, *BRAF*, or *Her2/neu* gene mutations and lack *TP53* mutations [23,24]. These tumors remain confined to the ovaries and constitute 25% of ovarian cancer. Tumors classified as Type 2, however, constitute 75% of ovarian carcinomas, are fast growing and are thought to evolve from the intraepithelial carcinoma in the Fallopian tube [25]. The vast majority are characterized by *TP53* mutations commonly known as the ‘*P53* signature’ and lack mutations of *KRAS*, *BRAF*, or *Her2/neu*. These tumors may exhibit gene amplification and over expression of the *HER2/neu* (10–20%) and *AKT2* (10–20%) oncogenes [23]. Gene expression profiles have shown Type 1 tumors to cluster separately from Type 2 tumors, suggesting that the two groups of tumors have a different genetic composition [25]. Figure 1 depicts the origin of Type 1 and Type 2 ovarian tumors.

## 4. What Is Known about Ovarian Cancer Progression?

As described above, ovarian cancer can arise from distinct progenitor cells; either from the cells of ovarian surface epithelium that has undergone neoplastic changes or from the Fallopian tube epithelial cells or oviduct fimbriae or from stem-like precursors of the junctional hilum region. The tumor microenvironment plays an essential role in the growth, invasion, and metastatic potential of malignant ovarian tumors and therefore represents an attractive therapeutic target [26,27]. Ascites (tumor fluid) is present in ~50% of advanced-stage ovarian cancer patients and is associated with a bad prognosis [26]. Ascites is accumulated in the peritoneal cavity due to the obstruction of the lymphatic vessels caused by the implantation of tumor cells preventing the outflow of fluid that transpires from the tumor vessels. Hence, in advanced-stage ovarian cancer patients the peritoneal cavity is invaded by metastatic tumors that grow as aggregates of floating cells commonly known as spheroids. It is believed that spheroids originate from single cells or sheets of tumor cells shed from the primary tumors on the ovarian surface epithelium or tumor cells that may be sloughed through the fimbriae of the Fallopian tube onto the ovarian surface epithelium as floating single cells or cellular aggregates in the ascites. In ascites, these multi-cellular spheroids may disaggregate and attach to the mesothelial lining of the peritoneum allowing the formation of secondary lesions [28,29]. Once adhered to the peritoneum, spheroid cells proliferate, migrate and invade to the surrounding tissues. In addition to the tumor cells, ~40% of the ovarian cancer ascites microenvironment consists of a variety of other cell types including stromal cells, immune cells, endothelial cells, as well as non-cellular components such as the extracellular matrix (ECM) components, remodeling enzymes (e.g., metzincins), metzincin inhibitors [e.g., TIMPs and reversion-inducing cysteine-rich protein with Kazal motifs (RECK) and growth factors like vascular endothelial growth factor (VEGF), transforming growth factor-β (TGF-β) and platelet derived growth factor (PDGF)] [29]. All these components create a microenvironment conducive for tumor cell growth, migration, invasion and metastasis.

Tumor cell plasticity is defined by two basic mechanisms: epithelial to mesenchymal transition (EMT), and the reverse, mesenchymal to epithelial transition (MET) [30,31]. EMT is a highly coordinated event, characterized by the transition of cells from an epithelial cobblestone-shape phenotype attached to a basement membrane to an elongated spindle-shaped mesenchymal morphology, accompanied by an increase in motility and invasive behavior [31]. Cells undergoing EMT may also resist anticancer therapies [32,33].

During normal ovarian function, modulation of ovarian surface epithelium to a fibroblastic phenotype by EMT has been shown to occur during post-ovulatory repair of the epithelium [6,34]. It has also been proposed that, EMT-transformed tumor cells are shed from the ovaries or sloughed through the fimbriae of the Fallopian tube into the peritoneal cavity where these cells undergo MET after subsequent attachment to the peritoneal/omental wall [5,28]. Furthermore, MET was postulated to also allow shed cells to proliferate in the peritoneal cavity as spheroids [34,35]. It has been suggested that hypoxic conditions in the tumors leads to the activation of several transcription factors resulting in EMT [36]. However, MET will occur when metastasized ovarian tumor cells encounter the new microenvironment in the peritoneal cavity, under normoxic conditions which allow the cancer cells to survive in the new environment [34].

ECM has been reported to affect the behavior of cancer cells by providing support and biochemical signals by growth factors and cytokines that are anchored or secreted from the ECM [36]. Some molecules are secreted by cells into the ECM to control different biological activities at the tissue or cellular level [37]. These include major members of the metzincin family, (MMPs, ADAMTSs, etc.) and their endogenous inhibitors (TIMPs), which have been implicated in ECM degradation and cell membrane receptor shedding for regulating cellular signaling and functions [38,39,40]. Together with TIMPs, metzincins constitute the major proteolytic axis of the ECM, and thus, are crucial for cancer progression [41].

## 5. Understanding the Structure and Roles of Metzincins and Their Inhibitors

### 5.1. Matrixin Family of Metzincins

#### Matrix Metalloproteinases (MMPs)

**Figure 2:**
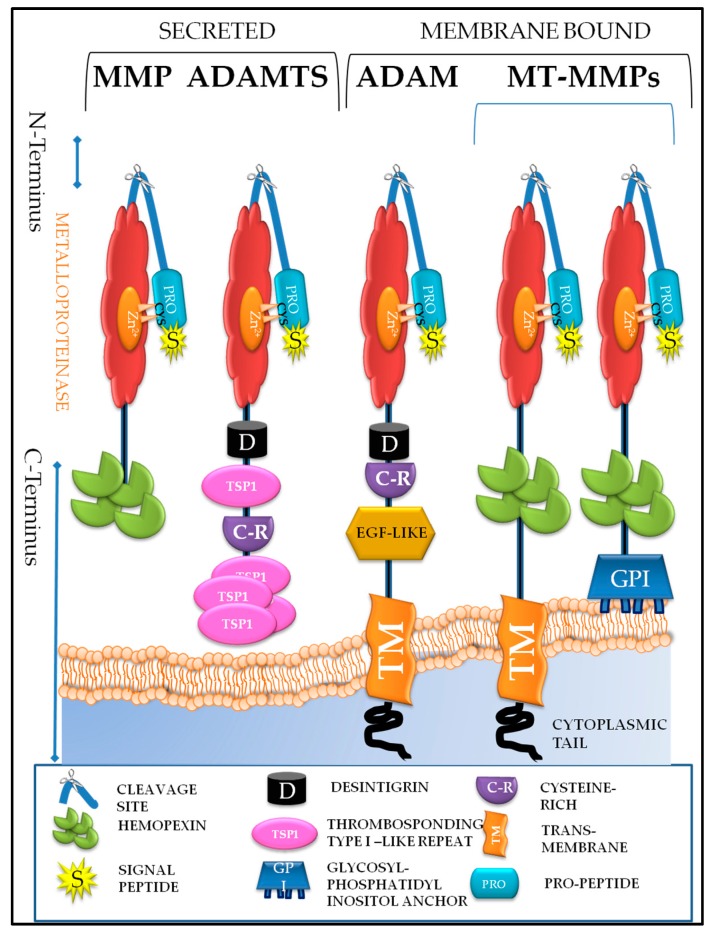
Schematic diagram of metzincins in human biology. The metzincin family members are characterized by their N-terminal domain structure. They have a signal peptide, a propeptide domain (containing a conserve ‘cysteine switch’), a flexible hinge region (cleavage site) and a zinc dependent catalytic domain (metalloproteinase). However, the C-terminal domain is what makes them unique; secreted MMPs contain a hemopexin-like domain that modulates substrate recognition. Membrane bound MMPs have either a GPI or a trans-membrane domain attached to a cytoplasmic tail that helps them to anchor to the plasma membrane of cells. ADAMs are membrane bound proteins that have disintegrin domain, a transmembrane domain, a cysteine-rich domain, and epidermal growth factor (EGF)-like domain and a cytoplasmic domain. ADAMTS also have a disintegrin domain. However, unlike ADAMs, they are secreted proteins and their disintegrin domain is linked to a central thrombospondin type I-like repeat, a cysteine-rich domain and varying numbers of thrombospondin repeats.

In humans, the MMPs are a family of 23 structurally and functionally related calcium dependent zinc-containing endoproteinases, which are involved in tissue remodeling and degradation of the ECM components including collagens, elastins, gelatin, matrix glycoproteins and proteoglycans between cells and in the lining of blood vessels [40,41,42,43]. In cancer, increased levels of MMPs degrade a variety of ECM component proteins, which allow the tumor cells to escape from their original location and disseminate enabling metastases. MMPs consist of a conserved N-terminal domain (a signal peptide, a pro-domain and a catalytic domain). The signal peptide targets the enzyme to the endoplasmic reticulum for transportation out of cells. The pro-domain contains a conserved cysteine-switch sequence (PRCGVPD) shielding the neighboring zinc dependent catalytic domain. The pro-domain is connected to the catalytic domain by a flexible hinge region, which is also known as the cleavage site. MMPs also contain a C-terminal (carboxyl-terminal) domain containing the hemopexin-like domain that modulates substrate recognition. The membrane-type MMPs [(MT-MMPs: MMP-14, 15, 16, 17, 24 and 25; cysteine array matrix metalloproteinase (CA-MMPs: 23A, 23B)] have additional transmembrane domains or glyosylphosphatidylinositol (GPI) anchors that bind them to the cell surface (Figure 2).

MMPs typically are secreted as inactive zymogens (pro-MMPs) in which the conserve cysteine residue in the pro-domain coordinates with the zinc ion in the active site and prevents binding and cleavage of the substrate, keeping the enzyme in an inactive form. Proteolytic cleavage of this pro-domain (at the cleavage site) by other MMPs or proteinases leads to the displacement of this pro-domain which allows for enzyme activation [40,41]. In vitro, pro-MMPs can be activated through modification of the thiol group of cysteine with physiological agents such as oxidants or disulphides or non-physiological agents for example, alkylating compounds or heavy metals, which can lead to irreversible activation of pro-MMPs [1].

MMPs are classified according to their substrate specificity and partly on their cellular localization. These groups are collagenases, gelatinases, stromolysins, matrilysin, and membrane type MMPs [43] (Table 1). Collagenases (MMP-1, MMP-8, MMP-13, MMP-18) can degrade and process collagen triple helix; stromolysin (MMP-3 and MMP-10) are structurally similar to collagenases but cannot degrade native collagen, although they can activate several pro-MMPs. The gelatinases (MMP-2 and MMP-9) have a fibronectin type II motif in their catalytic domain enabling them to process gelatin, while matrilysins (MMP-7 and MMP-26) are characterized by the lack of haemopexin domain [1]. The three furin activated proteinases (MMP-11, MMP-21 and MMP28) containing a furin recognition motif between the pro- and the catalytic domains are activated by intracellular furin-like proteases. The other group of furin activated proteinases are anchored to the plasma membrane of cells either through a GPI anchor (MMP-17 also known as MT4-MMP, and MMP-25 also known as MT6-MMP) or through an extensive (70–100 amino acid) hydrophobic type 1 transmembrane (MMP14, also known as MT1-MMP, MMP-15 also known as MT2-MMP, MMP-16 known as MT3-MMP and MMP-24 known as MT5-MMP) [1,40] (Figure 2).

In short, MMPs degrade ECM components to facilitate tissue remodeling during normal physiological processes including reproduction, embryogenesis, angiogenesis and cellular immunity. This facilitates cellular migration and release of cell-bound chemokines, cytokines and growth factors critical for cell–cell communication and signaling [43]. As MMPs have ECM degrading abilities, their regulation is tightly balanced at the transcriptional level, by the activation of inactive zymogens and inhibition by the endogenous proteinase inhibitors, such as TIMPs. Under normal physiological conditions, MMP activities are largely undetectable but their expression is enhanced during specific biological processes such as bone development, wound healing, angiogenesis and mammary involution [1].

### 5.2. Adamalysin Family of Metzincins

#### 5.2.1. A Disintegrin and A Metalloproteinases (ADAMs)

In humans, the ADAMs are a family of 22 known genes, encoding at least 12 proteolytically active functional proteins. These functional proteins are involved in tumor metastasis, angiogenesis, membrane fusion, cytokine and growth factor shedding and cell migration as well as processes such as fertilization, neurogenesis, myogenesis, embryonic TGF-α and epidermal growth factor receptor (EGFR) release and signaling [55,56,57,58,59,60,61,62,63,64,65,66,67,68,69,70,71,72,73,74,75,76,77,78,79,80,81]. ADAMs are membrane-anchored metalloproteinase that process and shed the ectodomains of membrane-anchored growth factors, cytokines and growth factor receptors [79,82,83,84,85]. ADAMs are mostly synthesized in the rough endoplasmic reticulum and mature in the late Golgi compartment [84,85,86]. Functionally active ADAMs proteins are usually detected on the cell surface [84,85,86,87].

ADAMs are made up of many different domains: an N-terminus domain (pro-peptide domain); a zinc dependent domain (metalloproteinase); a disintegrin domain, a transmembrane domain, a cysteine-rich domain, an epidermal growth factor (EGF)-like domain and a cytoplasmic domain (C-terminal tail) (Figure 2). The cytoplasmic domain helps ADAMs to anchor to the cell surface and to be involved in intracellular signaling [87,88]. The N-terminus of ADAMs contains a signal sequence that directs ADAMs into the secretory pathway and a pro-domain function in ADAMs maturation [87,88,89].

The shedding of extracellular domains of membrane-bound growth factors, cytokines and their receptors by ADAMs is essential for the activation of growth factors and cytokines that play crucial roles in essential cellular functions such as cell–cell adhesion, extracellular and intracellular signaling, cell differentiation and cell proliferation [78,85]. ADAM-10 for example, was reported to cleave the ectodomain of vascular endothelial (VE)-cadherin, a major adhesion molecule of endothelial adheren junctions [90]. This phenomenon is essential in controlling endothelial permeability, vascular integrity, leukocyte transmigration, and angiogenesis. Elevated levels of soluble VE-cadherin are associated with diseases like coronary atherosclerosis and cancer. In another example, the ectodomain shedding of epidermal growth factor receptor (EGFR) ligands, involved with the pathogenesis of hyper-proliferative disorder, such as cancer, has been shown to be contributed by ADAM-9, -10, -12 and -17 [91,92,93,94,95]. In addition, ADAM-17 has been shown to cleave the interleukin-6 receptor (IL-6R) resulting in the shedding of soluble interleukin-6 (sIL-6) which is a prerequisite for the pathogenesis of interleukin 6 (IL-6) trans-signaling effecting multiple cellular functions related to cancer and inflammation [96]. Consistent with that, an isoform of ADAM-8 found in lung tumor cell lines but not in normal cells, has been shown to contribute to tumor-derived bone metastasis through increased secretion of pro-osteoclastogenic cytokines IL-8 and IL-6 [97].

Recent studies have shown the interaction of ADAM proteins with integrins to play an important role in cancer progression [98]. ADAM-15 was shown to bind to αvβ3 integrin on the monocytic U937 cell line through the RGD-integrin binding sequence to facilitate cell ECM adhesion [99]. The expression of αvβ3 integrin on endothelial and tumor cells is regulated by vascular endothelial growth factor (VEGF) and is required for the metastasis of tumor cells to bone in prostate cancer [100]. ADAM-9 was reported to mediate the motility of cells by binding to α6β1 integrin [101]. ADAM-9 in human myeloma cells was further demonstrated to facilitate adhesion by binding to αvβ5 integrin, while the lymphoblastoid cells which do not express αvβ5 does not bind to ADAM-9 [102]. ADAM-9 was able to bind directly to α6β4 integrin on the surface of colon carcinoma cells through the disintegrin domain to promote carcinoma invasion [103]. In addition, ADAM-9 has been demonstrated to cleave L1 adhesion molecule, a protein which stimulates cell migration on fibronectin and laminin through mechanisms dependent on autocrine binding of L1 to αvβ5 integrin [104]. ADAM-9 also regulates myeloma cell induced ionterleukin-6 production by binding to αvβ5 integrin [105]. The metalloproteinase activity of particular ADAMs is inhibited by specific TIMPs (Table 1).

#### 5.2.2. ADAMS with Thrombospondin Motifs (ADAMTS)

The ADAMTS family contains 19 members [106]. They include enzymes involved in collagen biosynthesis as procollagen propeptidases (ADAMTS-2, ADAMTS-3 and ADAMTS-14) [107,108,109,110], the family of ‘aggrecanases’ responsible for the degradation of aggregan (ADAMTS-1, ADAMTS-4, ADAMTS-5, ADAMTS-8, ADAMTS-9 and ADAMTS-15), and specific aggrecanases that degrade the interglobular domain separating G1 and G2 of aggrecan at a specific Glu373–Ala374 bond (ADAMTS-11, and in some cases ADAMTS-4) [111,112,113,114,115].

In contrast to the ADAMs, the members of the ADAMTS protease family are secreted proteins. Structurally, ADAMTS consists of an N-terminus (propeptide) domain, a metalloproteinase domain and disintegrin domains (Figure 2). Furthermore, ADAMTS do not have a cysteine-rich domain, an epidermal growth-factor-like domain, or a cytoplasmic tail. Instead, the disintegrin domain is linked to a central thrombospondin type I-like repeat, a cysteine-rich domain and varying numbers of C-terminal thrombospondin repeats (Figure 2). ADAMTS-1, -4, -5, -8, -9, -15, -16, and -18 are regarded as proteoglycanases because they can degrade aggrecan, versican, brevican, and other proteoglycans. In contrast, ADAMTS-2 participates in the removal of the amino prodomain from procollagen I in the dermis [39]. Importantly, like MMPs, ADAMTS have many other functions, including processing and activating proteinase precursors, besides degrading the ECM (Table 1).

Aberrant methylation of *ADAMTS-1* has been shown to be involved in the development and progression of gastric cancer [114]. In vitro, ADAMTS-2, -3 and -14 have been shown to participate in processing of fibrillar procollagen types I–III, and *ADAMTS-2* mutations have been linked to Ehlers–Danlos syndrome, a connective tissue disorder characterized by impaired collagen assembly [115,116,117]. *ADAMTS-9* acts as a functional tumor suppressor in gastric cancer through inhibiting the oncogenic AKT/mammalian target of rapamycin (mTOR) signaling pathway and ADAMTS-9 is related to beta cell function in type 2 diabetes [118]. ADAMTS-13 regulates the process of coagulation in the circulation by cleaving von Willebrand factor (VWF) multimers into small, inactive fragments [119]. In addition, ADAMTS-4 and ADAMTS-5 are upregulated in arthritic disease and this may contribute to disease progression through the degradation of aggrecan [120,121,122].

## 6. What Are Metzincin Inhibitors?

### 6.1. Synthetic Inhibitors

Several small molecule synthetic inhibitors of metzincins have been discovered and these have been extensively reviewed in recent publications [123,124,125,126]. Despite the introduction of a large number of MMP inhibitors in clinical trials, only one compound (periostat) has been approved by US Food and Drug Administration (FDA) for the treatment of periodontal diseases [127]. The early clinical trials of several broad spectrum hydroxamate and non-hydroxamate-type inhibitors were followed by unsuccessful advanced clinical trials because of several muculoskeletal side-effects [123,128]. This failure of MMP inhibitors in clinical trials is mainly attributed to complex biology of MMPs which for example in cancer can have both cancer promoting effects while other isoforms of MMPs can counterbalance that by having anti-angiogeneic and anti-metastatic effects [123,129].

Among the ADAM family, inhibitors for ADAM-17, a protease that releases the pro-inflammatory cytokine tumor necrosis factor-a (TNF-α) from its membrane-bound precursor have been well studied [130,131]. There are strong evidences that ADAM-17 is a major TNF-α convertase (TACE). Since elevated levels of TNF-α is associated with inflammatory diseases like arthritis and Crohn’s disease majority of the small molecule inhibitors for TACE have been focused on these diseases [123,130,131]. Unfortunately, side effects like hepatoxicity and lack of efficacy led to the discontinuation of these inhibitors in clinic with only two inhibitors being considered for Phase 2 clinical trial [132]. ADAM-10 and ADAM-17 facilitate the shedding of multiple *ErbB* ligands, which are overexpressed in several cancers and promote tumorigenesis upon binding to *ErbB* receptors [133]. Small molecule inhibitor for ADAM-10 in conjunction with ADAM-17 has advanced to clinical trial but their structure still remains unknown.

To date no effective synthetic inhibitor for ADAMTS family has been discovered despite continual efforts to discover effective inhibitors for ADAMTS-4 and ADAMTS-5 which would control the aggrecanolytic activity associated with osteoarthritis [123]. Hence, future advance in this field is required to selectively block for better therapeutic impetus of ADAMTS family.

### 6.2. Endogenous Inhibitors

Three endogenous inhibitors are known to negatively regulate metzincin activity; these are: α2-macroglobulin (primary inhibitor in blood and lymphatic tissue), RECK and TIMPs [2]. RECK is a membrane anchored protein which has been shown to act as a tumor suppressor gene in gastric and other cancers [134,135]. The expression of RECK correlates inversely with a small number of metzincin proteases (e.g., MMP-2, MMP-9, MT1-MMP, ADAM-10, and ADAM-17) primarily in the context of tumorigenesis [2,135,136,137,138]. RECK expression is normally down regulated/suppressed in tumorigenic cells [135,136]. RECK has been shown to control metastases by signal transducer and activator of transcription 3 (STAT3)-dependent neoangiogenic switch [136]; and to reduce the tumorigenesis of gastric cancer by inhibiting ADAM-mediated Notch1 shedding and activation [135].

TIMPs, on the other hand, represent a family of secreted proteins which can act as principal endogenous metalloproteinase inhibitors (Table 1) [139,140]. In mammals, TIMPs belong to a family of four genes that share significant homology in their structure, but differ in several aspects which are responsible for important differences in their respective roles (Table 2). TIMPs contain 184–194 amino acids (21–28 kDa) with two distinctive domains: an N-terminal domain and a C-terminal domain [53]. The N-terminal domain is very homologous across all TIMPs. Overall, the four human TIMPs are about 40% identical in sequence to each other; TIMP-2 and TIMP-4 are 50% identical in sequence, whereas TIMP-1 is only 37 to 41% identical to the other TIMPs [139].

The N-terminal domain of TIMPs is responsible for their common inhibitory function by folding within itself and wedging into the active site of the metzincins (e.g., MMPs, adamalysin); whereas the C-terminal domain is involved in properties more specific to each TIMP (Table 1) [140,141]. For example, in the case of TIMP-3, the C-terminal domain is responsible for its unique ability to bind to the ECM [142] and in the case of TIMP2, the C-terminal domain plays a role in its high affinity binding to the tumor cell surface [143].

### 6.3. Do TIMPs Only Inhibit Metzincin’s Activity or Are They Independent of Metzincin?

A major role of TIMPs is to inhibit active MMPs, and due to structural similarities in the active site of MMPs and adamalysins, some ADAMs and ADAMTSs are also inhibited by TIMPs (Table 1). However, recent studies have in detail shown MMP-independent functions for TIMPs [160]. In this context, TIMP-1 has been shown to reduce the growth rate of human breast epithelial (MCF10A) cells by inducing cell cycle arrest at G1 phase. TIMP-1-mediated cell cycle arrest is associated with the downregulation of cyclin D1 and upregulation of p27^Kip1^, necessary for phosphorylation of the tumor suppressor retinoblastoma protein (RB) [161]. On the other hand, growth-promoting activity of TIMPs have been shown to regulate distinct signaling through mitogen activated protein kinase (MAPK), adenosine 3′,5′ monophosphate (cAMP)-protein kinase A and activation of *Ras* pathways [162,163,164].

TIMP-2 has been shown to mediate activation of signaling pathways by direct binding to cell surface receptors [165]. Competitive binding studies have identified α3β1 integrin as a putative receptor on endothelial cells for TIMP-2 binding [166]. α3β1 integrin-TIMP-2 binding initiate’s receptor tyrosine kinase inactivation through the protein tyrosine phosphatase, Shp-1, which suppresses cyclin D1 and de novo synthesis of cell cycle inhibitor, p27^Kip1^, resulting in the hypophosphorylation of tumor suppressor retinoblastoma protein (*pRB*) leading to G1 phase growth arrest of endothelial cells to inhibit angiogenesis [160,166,167]. In addition, TIMP-2 also binds to insulin-like growth factor-1 receptor (IGFR-1) on endothelial cells for the inhibition of angiogenesis [168], while TIMP-1 interacts with tetraspanin CD63, which associates with the β1 subunit of integrin receptors for the regulation of cell death pathways mediated by extracellular regulated kinase (ERK) [169]. TIMPs also regulate a variety of neurological processes, which includes, differentiation/arrest of neuronal cell growth, enhanced central nervous system myelination, protection of blood-brain barrier and oligodendrocyte differentiation [167,170] (Table 2).

### 6.4. What Is the Expression of TIMPs in Tumors?

TIMP-2 expression levels have been studied in several types of tumors as prognostic markers for disease progression. There is a growing consensus which supports the notion that TIMP-2 expression levels are reduced in a variety of human tumor settings and this is correlated with enhanced tumor progression [165]. In that scenario, the level of TIMP-2 expression has been shown to correlate inversely with the metastatic behavior and histological grade of malignant gliomas and glioma-derived cell lines [171,172]. Consistently, TIMP-2 expression levels were lower in clear cell renal carcinoma compared to control groups [173]. On the other hand, increased TIMP-2 levels in tumor tissues correlates with reduced tumor growth and enhanced sensitivity to chemotherapy. Enhanced TIMP-2 expression in the serum and tumor tissues have been associated with a better prognosis in non-small lung cancer (NSCLC) patients [174]. Consistent with that increased TIMP-2 expression has been positively correlated with enhanced survival in patients with endometrial and breast cancers [175,176]. On the contrary, high TIMP-2 expression was noted in lung adenocarcinomas and was associated with poor prognosis [177]. TIMP-2 expression in lung adenocarcinomas was significantly associated with the alteration of driving proto-oncogene tyrosine-protein kinase (*c-Src*) and phosphatidylinositol 3-kinase/protein kinase B (PI3-kinase/AKT pathways) [177].

High expression of TIMP-1 has been reported in hepatocellular carcinoma tissues associated with advanced TNM stage, intrahepatic metastasis, portal vein and vascular invasion [178]. Co-culture studies of liver cancer cells expressing high levels of TIMP-1 with fibroblasts extracted from normal liver demonstrated accelerated proliferation, migration and invasion of liver fibroblasts through activation of stromal derived factor-1/C-X-C chemokine receptor type 4 (SDF-1/*CXCR4*)/PI3K/AKT signaling pathways, indicating that TIMP-1 promotes hepatocellular carcinoma through activation of cancer associated fibroblasts [178]. Similarly, expression profiling of TIMP-1 in gastric cancer showed TIMP-1 expressing cells to be mainly tumor-associated myofibroblasts [179]. In contrast, reduced TIMP-3 expression due to promoter methylation has been reported in non-tumorous tissue of hepatocellular carcinoma [166]. Reduced expression of TIMP-3 in hepatocellular carcinomas was associated with reduced tumor differentiation and increased metastatic activity in HCC cell lines [180].

## 7. Metzincins and TIMPS in Ovarian Cancer

### 7.1. What Is the Evidence of the Involvement of MMPs and TIMPs in Ovarian Cancer?

#### 7.1.1. Expression in Tumors, Ascites and Blood

Proteolytic enzymes that degrade the ECM are implicated in all phases of ovarian cancer progression [181]. The expression and roles of MMPs have been studied extensively in ovarian cancer [182]. A meta-analysis of 956 ovarian cancer patients from 8 independent studies demonstrated that tumor-derived MMP-2 expression can predict a lower overall survival rate and could be used as an independent negative prognostic factor [183]. Over expression of MMP-2 in the peritoneal implants, but not in the primary tumors, of ovarian cancer patients have been related to a significant risk of death [184]. The expression of MMP-2 in the cystic fluid of serous and mucinous ovarian cancer patients was shown to be significantly elevated compared to borderline and benign ovarian tumors [185]. In another independent study, MMP-2 level was positively correlated with clinical stage and metastasis of ovarian cancer patients [186].

In a cohort of women with high grade serous carcinoma, an increased MMP-9 tumor expression, quantified by automated immunostaining, was associated with a higher risk of death but not with tumor progression; however, no correlation of protein expression with TIMP-2 and MMP-2 with death or tumor progression was observed in the same patient cohort [187]. Another study demonstrated no correlation between the expression of MMP-9 and TIMP-2 and survival in patients with advanced ovarian cancer [188]. The later study included 69 patients with the International Federation of Gynecology and Obstetrics (FIGO) stages III/IV, and the methodology included a tissue microarray/immunohistochemistry, with a visual histological scoring system to analyze the results [188]. In a third study an increase of TIMP-2 expression in the stroma and tumor compartment was positively correlated with a better survival rate of patients with ovarian cancer [189]. This study included 43 patient tumors (FIGO I (*n* = 1), FIGO II and III and grade 1–3 (*n* = 41); Serous (*n* = 37), endometriod (*n* = 3), and others (*n* = 3)). All analyses were performed using a visual immunoreactive scoring method (IRS). Hence, the conclusions from above three studies were different, possibly due the differences in the patient cohort, methodologies used for analyses and the different antibodies used to score the immunohistochemistry results.

High mRNA expression of MMP-2, MMP-9, MT1-MMP and TIMP-2 in primary ovarian tumors and stromal compartments was associated with poor survival in ovarian cancer patients, suggesting that MMP-2, MMP-9, MT1-MMP and TIMP-2 are valid markers of poor survival in advanced-stage ovarian carcinoma [190]. The epithelial expression of MMP-2, -7, -9, MT1-MMP, TIMP-2, but not TIMP-1, was reported to be higher in serous than mucinous ovarian tumors [191]. Stromal expression of MMP-7 was higher also in serous compared to mucinous tumors; while the expression of MT1-MMP, MMP-7 and -9 was similar in benign and borderline tumors compared to control group [191]. A recent study also showed higher expression of VEGF, MMPs, CD105 (Endoglin), TIMPs and VASH (Vasohibin) on advanced gynaecological cancers compared to healthy individuals [192]. A screening study using an orthotopic ovarian carcinoma model in nude mice recently demonstrated that the mRNA levels of TIMP-2, MMP-2 and other ovarian cancer associated proteins such as cytokeratin -7 (CK-7), cancer antigen-125 (CA-125), tumor suppressor *P53* and survivin, were significantly higher in cancer tissue than the middle paraneoplastic tissue and remote paraneoplastic tissue and normal ovarian tissue [193]. There was no statistically significant difference between the expression of these genes in middle and proximal paraneoplastic tissue as well as among residual normal ovarian tissues. Overall, the expression levels of CK-7, CA-125, *P53*, survivin, MMP-2, TIMP-2, and other molecular markers showed a decreasing trend in the non-cancer compared with cancer tissues.

In ovarian cancer, ascites rich in MMPs; MMP-2, -9, and -14 are major contributors to peri-cellular proteolysis in the peritoneal microenvironment [181]. Although proteinases are commonly expressed by stromal cells, epithelial expression of MMP-9 or MMP-14 in tumor cells has been correlated with decreased patient survival [181,194]. MMP-9 is expressed by primary ovarian carcinoma cells derived from the ovary, metastatic implants and ascites [194]. A recent study has revealed that MT1-MMP is one of the MMPs that contribute to the detachment of multicellular aggregates floating in the ascites microenvironment [195]. MMP-2 is also found to be abundantly expressed by the multicellular aggregates derived from ascites and is thought to play a major role in early metastasis by facilitating the rapid disaggregation of the ovarian cancer cells for adhesion to the mesothelial surface [196]. MMP-9 has also been reported to be highly expressed in the malignant mesothelium; specifically its expression was found to be stronger in mesothelial cells closer to the metastatic tumor and in mesothelial cells with a stratified and inflamed appearance, than those remote from the tumor [197].

Lysophosphatidic acid (LPA), a well characterized bioactive lipid molecule is abundantly present in the ascites and plasma of ovarian cancer patients [198,199]. LPA is constitutively produced by the mesothelial cells of the peritoneum, activates a G-protein coupled cell surface receptor and elicits uncontrolled proliferation, adhesion, migration, invasion and anoikis-resistance survival in floating ovarian cancer cells [200,201,202]. LPA has been shown to induce shedding of the 80 kDa extracellular domain of E-cadherin in ovarian cancer cells in a urokinase plasminogen activator (uPA)-dependent manner, disrupting cell-cell junctions and promoting EMT with enhanced motility and invasion [203]. In addition, LPA upregulates MMP-9 which also contributes to E-cadherin ectodomain shedding, contributing to EMT in ovarian cancer cells [204]. Activation of EGF receptor, which is frequently upregulated in ovarian cancer cells, also generates a ~80 kDa E-cadherin ectodomain fragment in ovarian tumor cells which contributes to ovarian cancer dissemination [205]. EGF-dependent down-regulation of E-cadherin was blocked by small interfering RNA (siRNA) specifically directed against MMP-9 and associations between EGF receptor activation, MMP-9 expression, and E-cadherin were evident in human ovarian tumors and paired peritoneal metastases [205,206]. The soluble E-cadherin ectodomain has been detected in peripheral blood, ascites and cystic fluids from ovarian cancer patients [207,208]. Furthermore, when this E-cadherin fragment was incubated with ovarian cancer cells at concentrations found in human ovarian cancer ascites, the fragment induced characteristics of EMT including altered morphology, disruption of cell–cell adhesion with loss of endogenous junctions and increased cell dispersion [206]. Above studies strongly suggest involvement of MMPs in the ectodomain shedding of E-cadherin which potentially regulates the EMT process in ovarian cancer.

Low serum TIMP-1 concentration at the end of first-line chemotherapy treatment in ovarian cancer was found to be associated with an improved survival rate of patients [209]. Microarray gene profiling on 6 advanced-stage high grade epithelial ovarian tumors before and after chemotherapy treatments found that tumor suppressor and apoptotic genes such as *SMOC2*, *TIMP-3*, *AXIN1*, *CASP4*, *P53* were significantly downregulated in post treatment tumors compared to chemonaive ones [210]. On the other hand, a recent study has reported that increased TIMP-2 expression in the stromal compartment, but not in tumor cells, is associated with an enhanced response to cisplatin and paclitaxel-based chemotherapy in ovarian cancer patients [189]. In addition, the expression of TIMP-3 was shown to be upregulated in recurrent ovarian tumors reactive stroma [211]. These results suggest that the suppression of TIMP-1 expression in the serum of post-chemotherapy patients is a good prognostic indicator for ovarian cancer patients. However, it is not clear, if similar suppressed TIMP-1 expression in tumors of post-chemotherapy patients may indicate the same prognosis. On the other hand, increase in TIMP-2 expression in the stromal compartment of tumors post-chemotherapy is a good indicator of cisplatin and paclitaxel responses in patients; and enhanced TIMP-3 expression in the stroma may be an indicator of recurrence in patients. These preliminary studies, however, need further validation for the proper use of TIMPs as biomarkers for ovarian cancer.

#### 7.1.2. Expression and Function in Cell Lines

Recent studies on ovarian cancer cell lines have demonstrated that the expression of metzincins in response to different stimuli can regulate the functions of ovarian cancer cells. LPA induced increased expression of MMP-1, also known as interstitial collagenase, in ovarian cancer cells have been correlated with increased invasion of cancer cells and has been shown to be mediated by the G-protein coupled receptor, protease-activated receptor-1(PAR1) [212,213]. Activation of MMP-1-PAR1 induces the secretion of interleukin-8 and growth related oncogene α from ovarian cancer cells, which act on endothelial chemokine CXCR1/2 receptors leading to endothelial cell proliferation, tube formation and cancer cell invasion [212,213]. Therefore, MMP-1-PAR1-CXCR1/2 pathways have been suggested as targets for ovarian cancer therapy. MMP-1 is also regulated by mixed lineage kinase 3 (MLK3) which is expressed at high levels in ovarian cancer cells [214]. MLK3 has been shown to activate mitogen-activated protein kinase (MAPK) signaling regulating proliferation, migration and apoptosis of ovarian cancer cells [214].

Enhanced expression of MMP-7, also known as matrilysin, in response to IL-8 and VEGF in ovarian cancer cells promotes invasion and metastasis of ovarian cancer through mesothelin (MSLN) activated MAPK/ERK and Janus kinase (JNK) pathways [215]. In addition, MMP-7 also promotes the invasion and metastasis of ovarian cancer through MSLN-activated MAPK/ERK and JNK pathways [216]. Hence, blocking MSLN-related pathway may be a potential target for ovarian cancer therapy.

EGF induced MMP-9 and TIMP-1 expression in ovarian cancer cell lines has been shown to result in increased migration and robust EGF-induced invasion [217]. A recent study examined the effects of the gonadotropin hormones, follicle stimulating hormone (FSH) and luteinizing hormone (LH), on ovarian cancer cell lines (BG-1, CAOV3, SKOV3 and OVCAR3) and observed a significant increase in their invasiveness. Both MMP-2 and MMP-9 were enhanced in one of the ovarian cancer cell lines (SKOV3) in response to gonadotropins, while TIMP-1 and TIMP-2 were down regulated, suggesting an inverse relationship between MMPs and their endogenous inhibitors [218]. The effect of docosahexanoic acid (DHA) on the invasiveness of A2780, HO8910, and SKOV3 cell lines was also investigated. DHA down regulated the expression of VEGF and MMP-9 and upregulated TIMP-1, kisspeptin-1 (KISS-1) and peroxisome proliferator-activated receptor gamma (PPAR-γ) which negatively correlated with cell invasion and metastasis associated gene expression in vitro. DHA restrained the development of sub intestinal vessels and cancer cell metastasis in a zebrafish xenograft model [219]. In other cell lines, SKOV3 treated with anti-inflammatory and anti-carcinogenic Bisdemethoxycurcumin (BDMC) was shown to inhibit MMP-2, MMP-9 and uPA expression but increase protein expression of TIMP-1 and reduced generation of cellular superoxide in a dose-dependent manner [220]. This occurred through reduced activation of the transcriptional protein complex, NFκB pathway suggesting, that BDMC can act as a therapeutic agent to suppress the aggressiveness of ovarian cancer cells. A recent study has shown extracellular vesicles shed from ovarian cancer cell lines to be rich in MMPs and their inhibitors, TIMPs, representing a mechanism of localized proteolytic activity to maintain cell–cell communication in the ovarian tumor microenvironment [221].

### 7.2. Adamalysin in Ovarian Cancer

High expression of ADAM-17 in early and advanced-stage ovarian tumors compared to normal ovaries has recently been reported; however, no clinical significance with progression-free survival was determined [222]. Contrary to that, an absent/low ADAM-12 tumor score was shown to correlate significantly with a shorter overall survival [223] suggesting that dysregulated ADAMs may govern ovarian malignancy. Genomics and clinical data from the Cancer Genome Atlas has demonstrated that somatic mutations of ADAMTS-1, -6, -8, -9, -15, -16, -18 were associated with higher sensitivity to platinum and longer progression-free survival, overall survival, and platinum-free survival duration in 512 patients with high-grade serous ovarian carcinoma [224]. Among the ADAMTS mutations, *ADAMTS-16* is the most commonly affected gene in ovarian cancer and exogenously expressed. *ADAMTS-16* missense mutations in A2780 cisplatin resistant cells inhibited cell growth and sensitized the cells to cisplatin and inhibited growth in vivo [224]. Orthotopic xenograft experiments from the same group showed that mice injected with ovarian cancer cells that exogenously expressed *ADAMTS-16* mutations had a better response to cisplatin treatment [224]. We have recently shown ADAMTS-1 to be enriched in ascites-derived chemoresistant/recurrent compared to chemonaive tumorigenic cells [225]. We also demonstrated that ADAMTS-9 mRNA is upregulated in chemoresistant/recurrent ascites-derived tumorigenic epithelial cells compared to chemonaive tumorigenic epithelial ascites cells [226]. In another study, a higher ADAMTS-1, ADAMTS-5, aggrecan, versican and TIMP-3 expression in malignant ovarian tumors compared to benign tumors was associated with a shorter overall survival in ovarian cancer patients [227]. As aggrecan and versican are the major ECM proteoglycan substrates used by ADAMTS-1 and ADAMTS-5, it is not surprising that their enhanced expression level correlated with increased expression of ADAMTS-1 and ADAMTS-5. However, it is not clear why there was a parallel increase in TIMP-3 expression, the tissue inhibitor of ADAMTS-1 and ADAMTS-5, in malignant ovarian neoplasms compared to benign epithelial ovarian neoplasm [227]. Consistent with these findings, investigation of poor survival signatures from three different serous ovarian cancer datasets identified TIMP-3 to be one of the prominent genes associated with poor survival rate [228].

### 7.3. What Is the Role of Metzincins in Ovarian Cancer? and What Are the Underlying Mechanisms?

Studies conducted over the past 40 years have provided mounting evidence supporting the role of metzincins in cancer [39,40,84,125,133]. Dysregulated expression of MMPs are known to affect cancer cell proliferation, apoptosis, anti-apoptosis, cell adhesion, migration, EMT, tumor angiogenesis, vasculogenesis as well as immune surveillance [39,40,41,82,133]. Relationship between MMPs with the occurrence and development of ovarian cancer and their underlying mechanisms has been discussed in detail in a recent review [182] and in Section 7.1 and Section 7.2 of this review.

The matrix remodeling role of MMPs in ovarian cancer is well studied and has been described in several recent studies [197,205,206]. Coordinated regulation of MMPs and TIMPs govern the cleavage and the release of many important growth factors and cell-surface receptors. One such example is TGF-β which plays a paradoxical role in cancer progression; it suppresses proliferation at early stages of cancer but promotes metastasis at late stages [229]. In vitro studies have shown that MMP-9 and MMP-2 can proteolytically cleave latent TGF-β, providing an important mechanism for TGF-β activation [230]. Another recent study has demonstrated that blocking ERK1/2 signaling impairs TGF-β1 tumor promoting function but enhances its tumor suppressing role in intrahepatic cholangiocarcinoma cells [231]. In this study TGF-β1 enhanced KKU-M213 cell invasion and migration and induced EMT as shown by the increase in vimentin, Slug and secreted MMP-9 levels and by a change in E-cadherin localization from membrane to cytosol. Aberrant TGF-β signaling has been reported in ovarian cancer [232] but whether TGF-β signaling in conjunction with metzincins; and more specifically with MMPs and cell proliferation exists in this tumor type still remains to be explored.

ADAMs on the other hand, are commonly known for their proteolytic activity required for the ectdomain shedding of growth factors and cytokines crucial for cell signaling associated with cancer cell proliferation, adhesion on ECM, migration and angiogenesis [78,82,83,133]. The role of different ADAMs is not extensively described in ovarian cancer but it can be postulated that their overall roles in cancers may apply broadly to ovarian cancer. It has been demonstrated that ADAM-10, which is overexpressed in both ovarian and uterine cancer, is involved with the ectodomain shedding of the adhesion molecule L1 through its autocrine binding with αvβ5 integrin [104]. Overexpression of ADAM-15 in ovarian cancer has been shown to disturb its interaction with αvβ3 interaction that resulted in reduced cellular adhesion to vitronectin and consequent reduction in cell motility [99].

Just like ADAMs and MMPs, ADAMTSs can also cleave or interact with a wide range of extracellular matrix components or regulatory factors, and therefore affect cell adhesion, migration, proliferation and angiogenesis. In conclusion, even though the expression of metzincins and their endogenous inhibitors in ovarian tumors, ascites, patient’s blood and ovarian cancer cell lines have been demonstrated; their overall contribution to ovarian tumor progression and recurrence still remains unclear. It is now clear that TIMP-signaling is complex and plays a pleotropic role in cancer biology. Hence, understanding the roles of metzincins and TIMPs is essential for the development of novel therapeutic interventions for ovarian cancer.

## 8. Chemotherapy Treatment Results in the Enhanced Expression of TIMP-1, -2 and -3 In Vitro and In Vivo: A Proof of Concept Experimental Model

We have previously demonstrated that the human ovarian HEY cell line treated with cisplatin or paclitaxel resulted in surviving residual population of cells which displayed a chemoresistant phenotype as evidenced by enhanced expression of DNA excision repair protein (*ERCC1*) and β-tubulin 3 compared to untreated control cells [33,233,234,235]. In addition, xenotransplantation studies using chemotherapy-treated surviving HEY cells generated significantly larger tumor burdens compared to untreated cells, and had a greater proliferative and tumorigenic capacity and retained an enhanced cancer stemness profile as evidenced by the enhanced expression of Ki67, CA-125, CD117 and Oct4 [33,233,234]. These results suggest that chemotherapy treatment leaves behind residual cells which have mechanisms for greater proliferation and self-renewal in vivo compared to untreated control cells.

**Figure 3:**
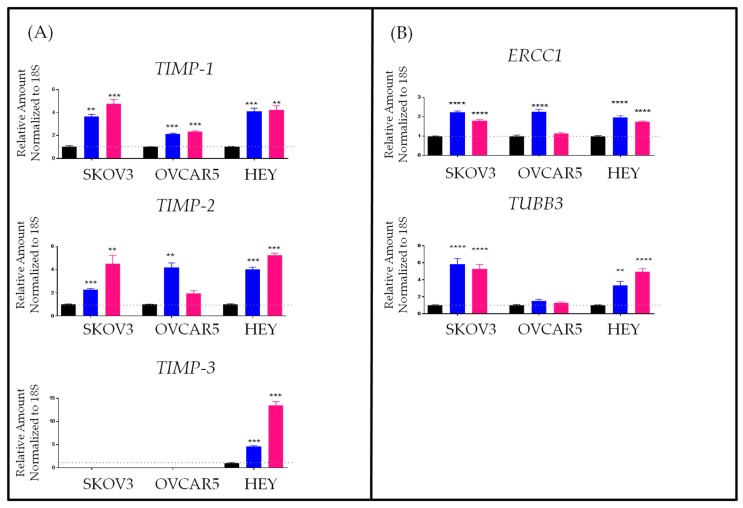
mRNA expression of *TIMP-1, TIMP-2, TIMP-3, ERCC-1* and *TUBB3A* in ovarian cancer cell lines treated with or without paclitaxel or cisplatin. (**A**) *TIMP-1*, *TIMP-2* and *TIMP-3* mRNA expression was deduced in (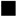) control SKOV3, OVCAR5 and HEY cell lines after treatment with IC50 doses of (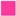) paclitaxel (0.67 µg/mL, 0.002 µg/mL and 0.0004 µg/mL respectively) and (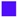) cisplatin (3.95 µg/mL, 4.57 µg/mL and 1.19 µg/mL respectively) for 72 h by qRT-PCR as described previously [235,238]. SKOV-3, OVCAR-5 and HEY cell lines have been described before [238]. The relative expression of gene of interest was normalized to house-keeping 18S gene. Data are shown as the mean ± SEM (*n* = 3). ** *p* < 0.01, *** *p* < 0.001. (**B**) The expression of ERCC1 and TUBB3A was performed in control, paclitaxel and cisplatin-treated ovarian cancer cell lines as described in Figure 3A. ** *p* < 0.01, **** *p* < 0.0001.

In this report, we demonstrate that treatment with chemotherapy (cisplatin or paclitaxel) resulted in significantly enhanced mRNA expressions of *TIMP-1, -2* in surviving residual cells compared to control untreated cells (Figure 3A). This was observed in three ovarian cancer cell lines SKOV3, OVCAR5 and HEY cells. However, *TIMP-3* was only elevated in HEY cells while SKOV3 and OVCAR5 cell lines had no mRNA expression of *TIMP-3* (Figure 3A). The enhanced expression of TIMPs in response to chemotherapy was consistent with significantly enhanced mRNA expression of genes associated with chemoresistance, *ERCC1* and class III β-tubulin (*TUBB3)*, in the surviving chemotherapy-treated cell lines (Figure 3B). We also provide preliminary data to demonstrate that this profile of chemoresistance in relation to TIMP expression can be retained in a mouse-xenograft model in which HEY cells were injected intraperitoneally. Nineteen days after injection of HEY cells (5 × 10^6^/mouse), mice were divided into three groups (*n* = 5/group). The first group of mice was control untreated, the second and third groups of mice received intraperitoneal injection of paclitaxel (15 mg/kg body weight) weekly. Treatment in all groups was maintained until the endpoint of control untreated mice was reached. At this point, mice in control and group 1 (paclitaxel-treated) were euthanized. Treatment in group 2 was terminated but the mice in this group were allowed to survive until the experimental end-point of each mouse was achieved (paclitaxel-recurrent). These mice survived two weeks longer than control untreated and paclitaxel-treated mice with end-point remaining the same as control mice [236].

Control untreated mice developed the largest tumor burden compared to all groups, with a mean of 13.6% ± 1.6% standardized to body weight [236]. There was a significant lower tumor burden in group 1 mice compared to control untreated mice, while the tumor burden in group 2 was similar to control group but significantly higher than the group 1 mice [236].

**Figure 4:**
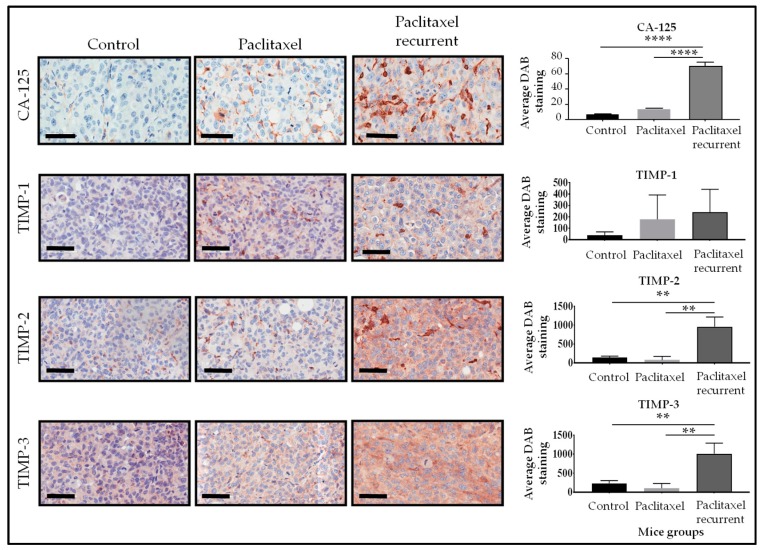
Representative images of TIMP-1, TIMP-2, TIMP-3 by immunohistochemistry staining in paraffin embedded tumor xenografts derived from mice in control, groups 1 and 2 was performed as described previously [33]. Animal experiment was carried out in strict accordance with the recommendations in the Guide for the Care and Use of the Laboratory Animals of the National Health and Medical Research Council of Australia. The experimental protocol was approved by the University of Melbourne’s Animal Ethics Committee (Project-1413207.1, 14 July 2014). Quantification of immunohistochemical staining of TIMP-1, -2 and -3 was performed as described previously [238]. Data is presented as mean ± SEM (*n* = 3 control mice, *n* = 3 xenografts from mice treated with paclitaxel, groups 1 and 2). Magnification 400×, scale bar = 60 µm. Significance is indicated by ** *p* <0.01; **** *p* <0.0001.

Immunohistochemistry analysis of mouse xenografts showed significant elevation in CA-125 staining in group 2 compared to group 1 and control groups (Figure 4). Significant elevation of TIMP-2 and TIMP-3 expressions was also observed in group 2 compared to control and group 1 mouse (Figure 4). These observations in in vitro cell lines and in animal models provides ‘proof of concept’ data which suggests that chemotherapy treatment may induce a ‘chemoresistant niche’ which protects chemoresistant cells from cell death by promoting a microenvironment appropriate for the survival of resistant cells. It can be postulated that TIMP-2 and TIMP-3 expression may have a role in that survival mechanism which enables the residual chemotherapy-treated cells to promote recurrence. In that context, the anti-apoptotic role of TIMP-2 through interaction with MTI-MMP and activation of Ras-ERK1/2 and AKT pathways that protects tumor cells has recently been described [237], and the expression of TIMP-3 has been shown to be elevated in recurrent tumors [211].

## 9. Potential New Therapeutic Approaches for TIMPs

TIMPs are known to inhibit a diverse range of metzincins, thus the use of TIMPs as therapeutic agents have been controversial due to its implication in affecting normal physiology regulated by metzincins. However, as our pilot data and other recent studies suggest enhanced expression of TIMPs may be one of the factors attributing to chemoresistance in the survival of ovarian cancer cells after chemotherapy treatment. Under that scenario, it may be crucial to inhibit TIMP expression in chemoresistant ovarian cells after chemotherapy treatment. Recently, a number of investigators have suggested that manipulating specific TIMP/metzincin interaction by engineering TIMP structurally can modulate future therapeutic approaches which may essentially limit tumorigenicity in vivo. For example, it has been shown that the selective inhibition of ADAM12 catalytic activity can result through the removal of the AB-loop in N-TIMP-2, which impairs the interaction of ADAM12 with ADAM17/TACE2, resulting in the loss of ADAM12 targeted cell surface shedding of growth factor receptors and gelatinases required for the tumorigenic phenotype of cancer cells [239]. In another example, TIMP-1 engineering resulted in functional incompetence of MT1-MMP (MMP-14) [240]. Under normal conditions, the wild-type TIMP-1 is unable to inhibit MT1-MMP. However, the insertion of a triple mutation at the N-terminus (V4A + P6V + T98L or N-TIMP-1^mt1^) of TIMP-1 effectively blocked the ectodomain of MT1-MMP, thus impairing its ability to activate MMP-2 and inhibited CD44 shedding and endothelial tubule formation in human vascular endothelial cells in a 3D fibrin gel model and also the ability to activate pro-MMP-2 [240]. In another study, ‘T1:TX’ chemiras developed by replacing the C-terminal domain in TIMP-1 with those of TIMP-2, -3, and -4 dramatically increased the affinity of TIMP-1 for some metzincin members [241]. Furthermore, these chimeras were able to suppress TNF-α and HB-EGF shedding in cell-based assays and had no growth-promoting activity in vitro. Instead, these chimeras inhibited cell migration and motility in several cancer cell lines [242]. Overall these findings suggest that engineering selective TIMPs with refined specificity against specific metzincin members may form the basis for developing novel therapeutics against cancer.

Maintaining MMP:TIMP balance is crucial for the maintenance of tissue homeostasis [165]. MMP:TIMP balance has been shown to contribute to tumor progression [242]. Recently, an unbalanced expression of MMP:TIMP axis genes in tumors were correlated with aberrant epigenotype in various gene promoters [243]. Correction of these malignant epigenotypes by nuclear modeling was shown to rebalance the tumorigenic gene expression profile resulting in altered tumor cell morphology, attenuation of migration and invasion in vitro and reduced tumor growth in vivo [243]. The cytotoxic effects of chemotherapy have also been shown to induce imbalance in MMP:TIMP balance which if not corrected results in tumor progression and recurrence. An imbalance between the serum MMP-9 and TIMP-2 was shown to predict disease progression in patients with metastatic renal cell carcinoma in response to sunitinib treatment [244]. Even though there was no significant difference in the serum levels of MMP-9 and TIMP-2 between the baseline and the time of progression, the MMP9:TIMP-2 ratio was significantly higher at the time of progression in non-responders versus responders in response to sunitinib [244].

Recent studies support the notion that TIMP-2 expression is suppressed in tumors compared to matching normal or benign tissues [165]. In that scenario, adding TIMP-2 to the tumor microenvironment seems a logistic approach to tackle tumorigenesis. Recent studies have utilized retroviral vector-mediated forced expression of TIMP-2 and Ala+TIMP-2, a TIMP-2 variant that is deficient in MMP inhibitory activity due to substitution of a single alanine residue to the N-terminus of TIMP-2 [165]. In a series of experiments, these studies elegantly demonstrated that forced expression of TIMP-2 and Ala-TIMP-2 selectively alters tyrosine phosphorylation of VEGFR-2 at residues directly implicated in endothelial cell proliferation and migration [245,246,247]. In addition, there was inhibition of phospholipase C-γ, Akt and endothelial nitric oxide synthase (eNOS), severely compromising endothelial function in relation to angiogenesis. In another model of TIMP-2 and Ala+TIMP-2 in human lung carcinoma cell line A549, TIMP-2 and Ala+TIMP-2-derived A549 cancer cell lines produced significantly suppressed tumor growth compared to empty vector controls as late as 40 days post tumor cell inoculation [248]. The suppression of tumor growth was associated with marked decrease in tumor microvascular density count (CD31+ or CD34+) of xenografts by immunohistochemistry as well as decrease in focal adhesion kinase and AKT activities suggesting suppression of angiogenesis, tumor cell migration and growth in TIMP-2 and Ala+TIMP-2 mice xenografts [248]. Transcriptional profiling of TIMP-2 and Ala+TIMP-2 tumor cells and xenografts demonstrated changes in cell-membrane association of E-cadherin and β-catenin, suggestive of mesenchymal to epithelial (MET) transition [248]. These studies in murine tumor models clearly suggest that either the exogenous administration of recombinant TIMP-2 or the use of nanoparticle-based delivery vehicle which can deliver TIMP-2 to tumor sites may have a potential for therapeutic utility in cancer where TIMP-2 is suppressed. In this context, lipid nanoparticles have shown great potential as delivery vehicles for a wide range of biomedical applications, including anti-cancer therapies. The inherent advantage of the nanoparticle-based delivery systems is that they tend to accumulate in tumors much more than in normal tissues. This occurs due to enhanced permeability and retention (EPR) effect due to the high density and defective vasculature as well as the lack of lymphatic drainage around tumors [249]. The administration of exogenous recombinant TIMP-2 may sustain normalization of tumor microenvironment by keeping the actions of metzincins under check which may support intact functional ECM and vascularization and may also facilitate a broader window for chemotherapy treatment response [151]. However, this will have to be improved by increasing the half-life of TIMPs, which in the case of murine TIMP-1 has been reported to have a plasma clearance of ~4 h [250]. Much of this can be improved by using modified forms of TIMPs, such as PEGylated TIMP-1, which has an extended half-life of 28 h in mice [250].

Few recent studies have shown the use of conditionally replicating adenoviruses (CRAd), which selectively kills tumor cells, loaded with *TIMP-2* as a therapeutic transgene, targeting the matrix metalloproteinase in murine orthotopic model of disseminated ovarian cancer [251,252]. These studies showed that *TIMP-2* armed CRAd delayed tumor growth in mice and significantly increased the survival compared to unarmed CRAd. This therapeutic effect was mediated by the inhibition of *MMP-9* [252]. These studies clearly support the translational potential of CRAd-*TIMP-2* for patients with advanced ovarian cancer.

## 10. Conclusions

Our understanding of the mechanisms of action of TIMPs in cancer as a ‘family of tumor regulators’ has significantly changed from a relatively simple concept that the essential role of TIMPs is the maintenance of homeostasis of the ECM by sustaining the balance of TIMPs to metzincins; to the understanding that TIMPs can have effects on tumor cell growth, metastasis, survival and response to chemotherapy independently of the metzincins.

**Figure 5:**
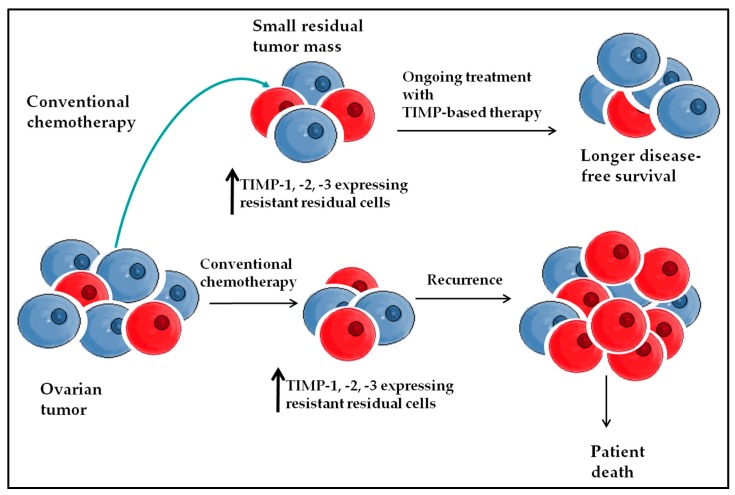
Proposed model of achieving a longer remission period with TIMP-based therapy in ovarian cancer. Current treatment of ovarian tumors after surgery with conventional chemotherapy results in smaller residual tumors containing chemoresistant cells. The surviving chemoresistant cells are able to proliferate; and subsequently develop recurrent tumors which lead to disease relapse and death in ovarian cancer patients. The addition of TIMP-based therapy may suppress TIMP expression in chemoresistant cells, which may suppress the growth of chemoresistant cells, thus impeding their tumorigenic potential, thus prolonging the overall remission period in ovarian cancer patients. 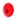 chemoresistant cells 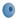 cancer cells.

The proof of concept data described in this report suggests that TIMPs may be involved in chemoresistance associated recurrence in ovarian cancer. These studies emphasize the need to evaluate the expression of metzincins and TIMPs in tumors in response to chemotherapy and other adjuvant therapies. This would facilitate detailed understanding of metzincins and their endogenous inhibitors in ovarian cancer biology; which in future could serve as a basis for the development of new diagnostic/screening and therapeutic approaches. This could potentially lead to a good response to cancer treatment, and thus increase survival of ovarian cancer patients. A model of TIMP-based therapy in advanced ovarian cancer is discussed in Figure 5.

## Figures and Tables

**Table 1:** Characteristics of metzincin family members known to be inhibited by TIMPs.

Class	Gene	Inhibited by	Location	Targets	References
Collagenases	*MMP-1*	*TIMP-1* *TIMP-2* *TIMP-3* *TIMP-4*	secreted	Substrates include Col I, II, III, VII, VIII and X; gelatin, *MMP-2*, *MMP-9*, proteoglycans, aggrecan, casein, serpins, versican, pro*TNF*-α, *IGF-BP-3*, *IGF-BP-5*, *IL-1*β, *SDF-1*, *PAR1*, *CD49b*, binds α2β1-integrin	[43,44,45,46,47,48,49,50,51,52,53]
	*MMP-8*	*TIMP-1* *TIMP-2* *TIMP-4*	secreted	Substrates include Col I, II, III, VII, VIII, X, aggrecan, gelatin, laminin, nidogen, proteoglycans, *ADAMTS-1*, pro-*MMP-8*, pro*TNF*-α, α2-antiplasmin, *IGF-BP*.	[43,44,45,46,47]
	*MMP-13*	*TIMP-1* *TIMP-2* *TIMP-3* *TIMP-4*	secreted	Substrates include Col I, II, III, IV, IX, X, XIV, gelatin, laminin, proteoglycans, aggrecans, fibronectin, pro *TNF*-α, pro-*MMP-9* and pro-*MMP-13*, *SDF-1*.	[43,44,45,46,47,48,49,50,51,52,53]
Gelatinases	*MMP-2*	*TIMP-1* *TIMP-2* *TIMP-3* *TIMP-4*	secreted	Substrates include Gelatin, Col I, II, III, IV, V, VII, X, XI, XIV, fibronectin, laminin-5, elastin, aggrecan, versican, active *MMP-9*, active *MMP-13*, *SDF-1*, *IGF-BP*s, *IL-1*β, pro-*TGF*-β1,*CXCL6*, α1-antyproteinase.	[43,44,45,46,47,53]
	*MMP-9*	*TIMP-1* *TIMP-2* *TIMP-3* *TIMP-4*	secreted	Substrates include Gelatin, Col IV, V, VII, X, elastin, laminin, versican, aggrecan, fibronectin, vitronectin, *CXCL5*, *IL-1*β, pro-*TGF*-β1, plasminogen, pro-*TNF*-α, *SDF-1*, *CXCL6*.	[43,44,45,46,47,53]
Stromelysins	*MMP-3*	*TIMP-1* *TIMP-2* *TIMP-3* *TIMP-4*	Secreted	Substrates include Col II, III, IV, IX, X, XI, gelatin, fibronectin, laminin, proteoglycan, versican, pro-*MMP-1*, pro-*MMP-7*, pro-*MMP-8*, pro-*MMP-9*, pro-*MMP-13*, pro-*TNF*-α, E-cadherin, l-selectin, *SDF-1*, pro-*HB-EGF*, pro-*IL-1*β.	[45,46,47]
	*MMP-10*	*TIMP-1* *TIMP-2*	Secreted	Substrates include Col I, III, IV, V, laminin, casein, pro-*MMP-1*, pro-*MMP-8*, pro-*MMP-10*, fibronectin, proteoglycans, elastin, gelatin	[45,46,47]
	*MMP-11*	*TIMP-1*	secreted	Substrates include Col IV, fibronectin, laminin, gelatin, aggrecan, α1-antitrypsin, α1-proteinase inhibitor, *IGF-BP-1*	[45,46,47,51]
Matrylisin	*MMP-7*	*TIMP-1* *TIMP-2* *TIMP-3* *TIMP-4*	Secreted	Substrates include: Col I, IV, X, fibronectin, laminin, gelatin, elastin, aggrecan, casein, proteoglycans, pro-*MMP-1*, pro-*MMP-2*, pro-*MMP-7*, pro-*MMP-8*, pro-*TNF*-α, E-cadherin, Fas-L, β4 integrin	[45,46,47,48,49,50,51,52,53]
	*MMP-26*	*TIMP-4*	secreted	substrates include: Col I, IV, fibrogen, fibronectin, laminin, gelatin, casein, elastin, proteoglycans, pro-*MMP-2*, pro-*TNF*-α, E-cadherin	[45,54]
Membrane Type-MMPs	*MMP-14*	*TIMP-2* *TIMP-3* *TIMP-4*	membrane-associated	Substrates include: Col I, II, III, gelatin, fibronectin, laminin, vitronectin, proteoglycans, tenascin, pro-*MMP-2*, pro-*MMP-13*, *SDF-1*, pro-*TNF*-α, αvβ3 integrin	[43,44,45,46,47,48,49,50,51,52,53]
	*MMP-15*	*TIMP-2* *TIMP-3* *TIMP-4*	membrane-associated	Substrates include gelatin, Col I, fibronectin, laminin, nidogen, tenascin, pro-*MMP-2*, pro-*MMP-13*, pro-*TNF*-α	[43,44,45,46,47,48,49,50,51,52,53]
	*MMP-16*	*TIMP-2* *TIMP-4*	membrane-associated	substrates include Col I, gelatin, fibronectin, laminin, vitronectin, aggrecan, casein, pro-*MMP-2*, pro-*MMP-13*	[43,44,45,46,47,48,49,50,51,52,53]
Metalloelastase	*MMP-12*	*TIMP-1* *TIMP-4*	secreted	Substrates include elastin, fibronectin, Col IV, gelatin, proteoglycans, plasminogen, laminin	[43,44,45,46,47,48,49,50,51,52,53]
Other MMPs	*MMP-19*	*TIMP-2* *TIMP-4*	–	Substrates include: Col I, IV, gelatin, aggrecan, casein, tenascin, nidogen, laminin.	[47]
Active ADAMs	*ADAM-10*	*TIMP-1* *TIMP-3*	membrane-associated	integrins CD11b/CD18 (Mac-1), junctional adhesion molecule (JAM)-A; Notch; *EGFR* ligands: *HB-EGF*; *EGF* and betacellulin; APP; Notch2, Notch3, N-, and E-cadherin; *CD23*, CD30, CD44, DLL1, Fas-L, HER2, L1, *TNF*-α Notch receptor, ephrin A5, collagen IV., *CXCL16*, nectin-4	[55,56,57,58,59,60,61,62,63,64]
	*ADAM-12*	*TIMP-1* (weak) *TIMP-2* *TIMP-3*	membrane-associated	gelatinase; *HB-EGF*; collagen IV, DLL1, fibronectin; *IGF-BP3*, *IGF-BP5*, *ADAM-10*, Betacellulin	[55,58,65,66,67]
	*ADAM-15*	*TIMP-3*	membrane-associated	*EGFR* ligands: *HB-EGF*; amphiregulin, *CD23*, collagen IV, E-cadherin, *ADAM-10*, gelatin	[55,60,68,69,70]
	*ADAM-17*	*TIMP-3* *TIMP-4*	membrane-associated	*TACE* (*TNF*-α converting enzyme); Mac-1, JAM-A; *EGFR* ligands, *HB-EGF* and amphiregulin; APP; EpCAM and ErbB4; CD44, collagen XVII, DLL1, epiregulin, epigen, ICAM-1, L-selectin, *Notch1*, transforming growth factor α (*TGF*-α), *TNF*-α, V-CAM1; mucin-1 (MUC-1), tumor necrosis factor receptor (*TNFR*)I, *TNFR*II, *IL-6R*, HER-4, vascular endothelial growth factor receptor 2 (*VEGFR2*), nectin-4	[55,63,71,72,73,74,75,76]
	*ADAM-28*	*TIMP-3* *TIMP-4*	membrane-associated	IGBP3, VWF, *CD23*	[55,76]
	*ADAM-33*	*TIMP-3* *TIMP-4*	membrane-associated	*IL-18*, APP, KL-1, insulin B chain	[55,77,78,79]
ADAMTS	*ADAMTS1*	*TIMP-2* *TIMP-3*	secreted	Substrates include: Aggrecan, versican, syndecan 4, TFPI-2, semaphorin 3C, nidogen-1, -2, desmocollin-3, dystroglycan, mac-2, gelatin (denatured collagen type I), amphiregulin, *TGF*-α, heparin-binding *EGF*	[80,81]
	*ADAMTS-2*	*TIMP-3* (weak)	secreted	Substrates include: Fibrillar procollagens types I-III and V	[80,81]
	*ADAMTS-4*	*TIMP-1* *TIMP-3* *TIMP-4*	secreted	Substrates include: Aggrecan, versican, reelin, biglycan, brevican, matrilin-3, α2-macroglobulin, Cartilage oligomeric protein (COMP)	[80,81]
	*ADAMTS-5*	*TIMP-3* *TIMP-4* (weak)	secreted	Substrates include: Aggrecan, versican, reelin, biglycan, matrilin-4, brevican, α2-macroglobulin	[80,81]
	*ADAMTS-9*	*TIMP-3*	secreted	Substrates include: Aggrecan, versican	[80,81]

**Table 2:** Characteristics of tissue inhibitors of matrix metalloproteinases.

Characteristic	*TIMP-1*	*TIMP-2*	*TIMP-3*	*TIMP-4*
Mol. weight (kDa)	28	21	24	22
Chromosome (human)	X11p11.23–11.4	17q23–25	22q12.1–q13.2	3q25
mRNA (kb)	0.9	1.2, 1.7, 3.5	2.4, 2.8, 4.5	1.4
Glycosylation	Yes	No	Partial	No
Amino acids (mature protein)	184	194	188	194
Binding	Pro-MMP-9, cell surface	Pro-*MMP-2*, cell surface	ECM, Pro-MMP-9 and pro-MMP2	Pro-MMP-2
Apoptotic effects	Negative (Suppresses)	Positive(Stimulates) Negative (Protects)	Positive (Enhances)	Positive(Stimulates) Negative (Protects)
Cell Growth	Stimulates	Stimulates Suppresses	Suppresses	No effect
Angiogenesis	Mediate through interaction with β1 integrin and CD63	Negative	Negative	Negative
Other Genes known to block			*VEGFR2*, *VEGF*	
Down-regulated by	STAT3, IL-4	*EZH2*, *SOCS1*, Baicalein (drug), Rosuvastatin (cholesterol lowering drug)	TNF, IL-4,	*TGF*-β1, LPS, *TNF* (weak), IL-4 (weak)
Upregulated by	*TGF*-β1, LPS, c-Jun	*TGF*-β1, TET1, angiotensin-II	*TGF*-β1, LPS	
References	[144,145,146,147,148,149]	[144,146,148,150,151,152,153,154]	[144,145,146,148,154,155,156,157,158,159]	[144,145,146,147,148]
